# The role of allergen-specific IgE in predicting allergic symptoms on dog and cat exposure among Korean pet exhibition participants

**DOI:** 10.1016/j.waojou.2020.100488

**Published:** 2020-11-27

**Authors:** Sung-Yoon Kang, Min-Suk Yang, So-Young Park, Jung-Hyun Kim, Ha-Kyeong Won, Oh Young Kwon, Ji-Hyang Lee, Ye-Won Kang, Jae-Woo Jung, Woo-Jung Song, Sae-Hoon Kim, Sang Min Lee, Sang Pyo Lee

**Affiliations:** a Department of Internal Medicine, Gachon University Gil Medical Center, Incheon, Republic of Korea; b Department of Internal Medicine, SMG-SNU Boramae Medical Center, Seoul, Republic of Korea; c Division of Pulmonary, Allergy and Critical Care Medicine, Department of Internal Medicine, Konkuk University School of Medicine, Seoul, Republic of Korea; d Department of Internal Medicine, Armed Forces Capital Hospital, Seongnam, Republic of Korea; e Department of Internal Medicine, Veterans Health Service Medical Center, Seoul, Republic of Korea; f DreamKwon Internal Medicine Allergy Clinic, Seoul, Republic of Korea; g Department of Allergy and Clinical Immunology, University of Ulsan College of Medicine, Asan Medical Center, Seoul, Republic of Korea; h Department of Internal Medicine, Pusan National University School of Medicine, Pusan National University Hospital, Busan, Republic of Korea; i Department of Internal Medicine, Chung-Ang University College of Medicine, Seoul, Republic of Korea; j Department of Internal Medicine, Seoul National University Bundang Hospital, Seongnam, Republic of Korea

**Keywords:** Allergy, Cats, Dogs, Skin prick test, Specific IgE, SPT, Skin prick test, sIgE, Allergen-specific IgE, MWD, Mean wheal diameter, A/H ratio, Allergen-to-histamine ratio, PPV, Positive predictive value, NPV, Negative predictive value, SN, Sensitivity, SP, Specificity, ROC, Receiver-operating characteristic, AUC, Area under the curve

## Abstract

**Background:**

The values of the skin prick test (SPT) and allergen-specific IgE (sIgE) measurement in predicting dog and cat allergies remain unclear. We aimed to evaluate the usefulness of SPT and sIgE measurement in predicting self-reported allergic symptoms during exposure to dogs and cats in Korean adults.

**Methods:**

A total of 552 participants in a pet exhibition in Korea completed questionnaires regarding exposure to dog or cat and the development of allergic symptoms during exposure. Study participants also underwent SPT using 3 different commercially available reagents, and had their blood drawn for measurement of serum total IgE and dog/cat-dander-IgE using ImmunoCAP®.

**Results:**

Measurement of sIgE for dog and cat dander allergens provided the highest positive and negative predictive values and sensitivity, but not specificity (58%, 87.2%, 67.9%, and 93.1% for allergic symptoms on dog exposure; 64.7%, 83.2%, 74.8%, and 88.9% for those on cat exposure, respectively), in predicting self-reported allergic symptoms on dog and cat exposure. The sIgE level consistently exhibited the highest area under the receiver operating characteristic curve (0.749 and 0.719 for allergic symptoms on dog and cat exposure, respectively). Careful interpretation of SPT and sIgE measurements maximized the positive and negative predictive values, sensitivity, and specificity for predicting allergic symptoms on dog exposure (71.4%, 87.3%, 75.3%, and 99.3%) and those on cat exposure (71.4%, 85.3%, 79.3%, and 98.9%).

**Conclusions:**

The measurement of dog and cat dander sIgE levels may be useful for the exclusion of allergic symptoms related to pet exposure. Collective interpretation of SPT and sIgE tests facilitates identification of allergic symptoms on dog or cat exposure, giving a better rule-in test result.

## Introduction

Domestic animals are common sources of allergens worldwide, and exposure to animal allergens is a major risk factor for allergic sensitization and subsequent development of allergic diseases.^1^^,^^2^ The number of pets per household, including dogs and cats, has also increased over the past decade. Furthermore, sensitization to pet allergens has also increased among patients with various *allergic* diseases.^3^ Animal allergens are small particles that can be easily spread and inhaled by humans; hence, the possibility of direct or indirect exposure to animal allergens is high, particularly among pet owners.^4^ Although allergen avoidance is recommended whenever possible, sufficient symptom control cannot be achieved with allergen avoidance alone; appropriate drugs or immunotherapeutic interventions for symptom relief are often required for effective control of allergic diseases.^5^, ^6^, ^7^ In addition to proper management of allergic diseases, the accurate diagnosis of allergies is crucial.

Skin prick test (SPT) and measurement of allergen-specific IgE (sIgE) level have been widely performed for allergy diagnosis and monitoring.^8^, ^9^, ^10^, ^11^ SPT is a rapid method that provides information on the sensitivity to individual allergens and therefore is used to diagnose respiratory allergic diseases. However, the accuracy of SPT can be influenced by various factors, including age, sex, race, concomitant drug treatments, and the SPT technique used.^12^, ^13^, ^14^ Allergy diagnosis can also be achieved by *in vitro* measurement of the sIgE level. Measurement of the sIgE level allows quantification and is not influenced by the operator technique, antihistamine intake, or underlying medical conditions.^15^

Since the introduction of these 2 methods into clinical practice in the late 20th century, their diagnostic performance has been evaluated and compared extensively.^8^^,^^16^, ^17^, ^18^, ^19^, ^20^ Nevertheless, their diagnostic value has not been established yet, especially for allergies to dogs and cats. In a previous study, only 38.8% of dog owners who suffered from allergic symptoms during exposure to their dog showed positive results for dog allergy in SPT, so did 31.1% of cat owners for cat allergy.^21^ In this study, using all 3 allergen extracts for SPT commercially available in Korea, we next planned to evaluate comprehensively the usefulness of SPT, as well as that of sIgE measurement, in predicting self-reported allergic symptoms on dog and cat exposure.

## Methods

### Study subjects

The study cohort included individuals who attended the “Korea Pet (KOPET) Show” pet exhibition from September 7–9, 2018. Individuals who were exposed to dogs and/or cats completed questionnaires regarding their exposure to these animals and the development of any allergic symptoms during exposure. Participants who had dermographism or who had administered medications that could influence the SPT results such as systemic glucocorticosteroids and tricyclic antidepressants, as well as antihistamines within a week were excluded from this study. These individuals underwent SPT for dog and cat allergens, and blood samples were collected to measure total IgE and sIgE levels induced by dog and cat dander allergens. Informed consent was obtained from all study participants.

### Questionnaires regarding demographics, pet ownership, and allergic symptoms on pet exposure

Study participants completed questionnaires regarding their age, sex, presence of allergic diseases, family history of allergic diseases, development of allergic symptoms during exposure to pets, and location of pet exposure (their own home, home of relatives or friends, workplace, or public places such as pet shops, pet café, and pet hospitals). The questionnaires were modified versions of the questionnaires administered to subjects in a previous study*.*^21^ Individuals who experienced any allergic symptoms during exposure to dogs or cats were considered as symptomatic group to dogs or cats. For this study, we classified allergic symptoms on dog and cat exposure into several categories, including allergic conjunctivitis, allergic rhinitis, asthma, skin allergy, and cough. We defined allergic symptoms as follows, with a temporal relationship between the appearance of those symptoms and pet exposure: 1) those who had allergic conjunctivitis, when the subjects suffered from red, itchy eyes, edema and profuse watery discharge; 2) those who had allergic rhinitis, when the subjects suffered from one or more of watery rhinorrhea, sneezing, nasal congestion, postnasal drip, and itchy nose apart from *common cold* or flu; 3) those who had asthma, when the subjects suffered from dyspnea, chest discomfort and wheezing that fluctuated with nocturnal aggravation; 4) those who had skin allergy, when the subjects suffered from one or more itchy hives and rashes on their skin; and 5) those who had cough, when the subjects suffered from recurrent or long standing cough.

### Skin prick test

Participants underwent SPT using dog and cat dander allergen extracts from 3 different companies (HollisterStier, Spokane, WA, USA; Lofarma, Milan, Italy; and Allergy Therapeutics, Worthing, West Sussex, UK). SPT was performed on the forearms by trained investigators using sharp-pointed lancets. Histamine solutions (10 mg/mL, HollisterStier; 1%, Lofarma; 0.1%, Allergy Therapeutics) and glycerinated saline were used as positive and negative controls, respectively. The skin test results were interpreted after 15 min by measuring the mean wheal diameter (MWD) induced by each allergen. The SPT results were regarded as positive according to 1 of 3 different conditions: a wheal of any size was induced by an allergen, the MWD was ≥3 mm, or the allergen-to-histamine ratio (A/H ratio) of MWD was ≥1.

### Serologic analysis

Blood samples were collected, and plasma was isolated for sIgE analysis. IgE antibodies against dog dander extract (e5) and cat dander extract (e1) were analyzed using the ImmunoCAP® system (Thermo Fisher Scientiﬁc, Uppsala, Sweden) according to the manufacturer's instructions.^22^^,^^23^ Results were presented as kilounits of allergen per liter (kUA/L); the cut-off values used to determine the presence of allergen-speciﬁc IgE were 0.10, 0.35, and 3.5 kUA/L as described in previous studies.^24^, ^25^, ^26^

### Statistical analysis

Continuous variables are expressed as means ± standard deviation or as medians (interquartile ranges), depending on their distribution. Categorical variables are expressed as absolute numbers and percentages. For continuous data, comparisons between groups were performed using Student's *t*-test (parametric data) or the Mann–Whitney *U* test (non-parametric data). For categorical data, comparisons between groups were performed using the chi-squared test. The positive predictive value (PPV), negative predictive value (NPV), sensitivity (SN), specificity (SP), and receiver operating characteristics (ROC) curve analysis, summarized by area under the curve (AUC) with 95% confidence intervals (CIs), were obtained to assess the diagnostic performance of the tests. All statistical analyses were performed using SPSS version 18.0 (SPSS Inc., Chicago, IL, USA). Two-sided *p* values less than 0.05 were considered statistically significant.

## Results

### Study population

**Fig. 1:**
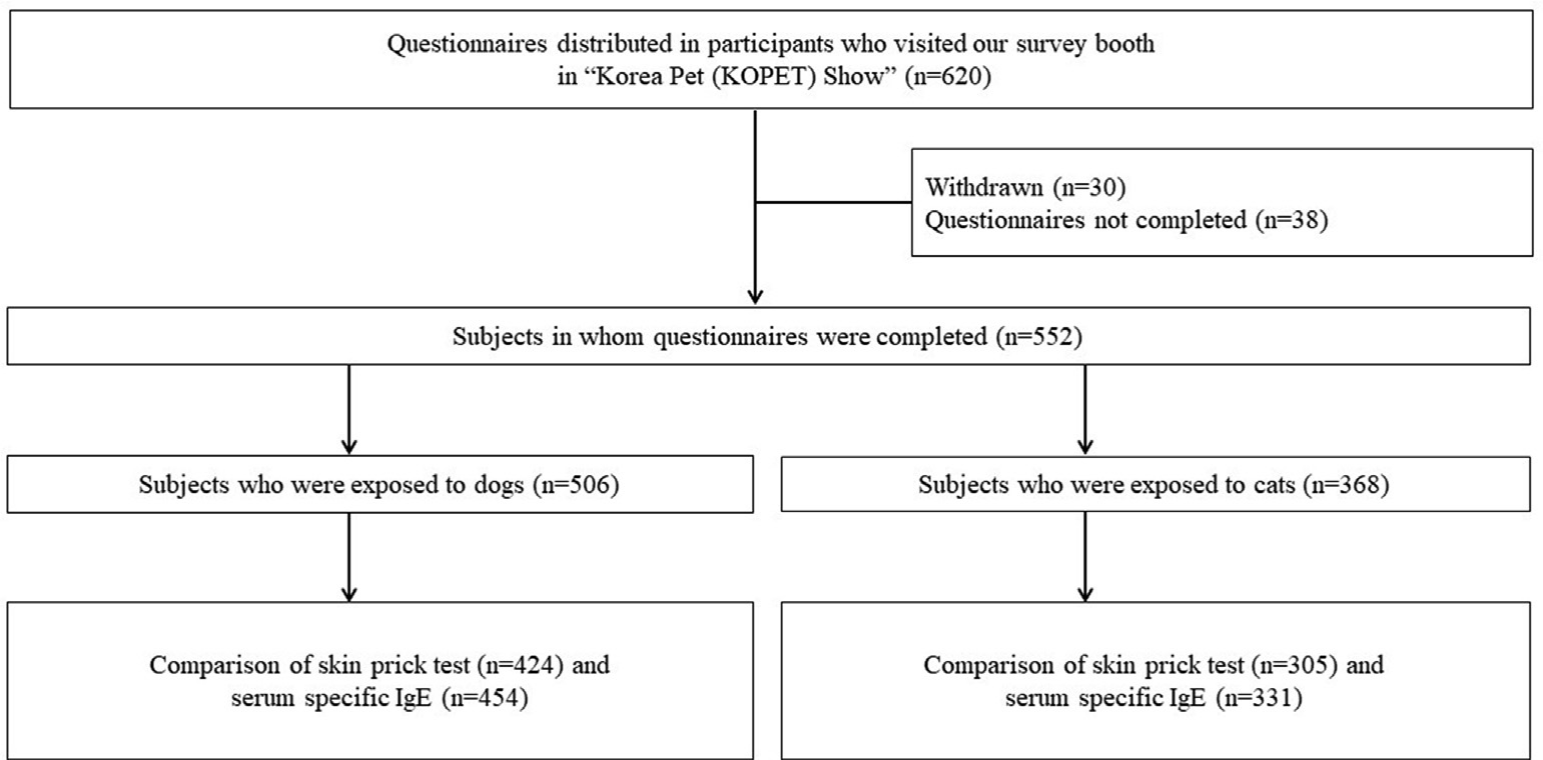
Flowchart of the study population.

Initially, 620 KOPET exhibition participants were enrolled in our study. Of these participants, 30 (4.8%) eventually withdrew from the study, and 38 (6.1%) were excluded due to incomplete questionnaires. Thus, our analyses included data from 552 (89.1%) individuals (Fig. 1). The demographic characteristics of the study participants are shown in Table 1.

**Table 1:** Demographic characteristics of the study subjects.

	All subjects (n = 552)	Subjects with exposure to dogs (n = 506)			Subjects with exposure to cats (n = 368)		
		Symptomatic group^a^ (n = 112)	Non-symptomatic group (n = 394)	*P*-value∗	Symptomatic group^a^ (n = 125)	Non-symptomatic group (n = 243)	*P*-value∗
Female	439 (79.5)	88 (78.6)	317 (80.5)	0.660	104 (83.2)	192 (79.0)	0.338
Age, years	30.2 ± 9.2	30.5 ± 8.7	29.8 ± 9.3	0.447	29.1 ± 8.1	28.5 ± 8.2	0.535
Underlying allergic diseases							
Allergic rhinitis	245 (44.4)	80 (71.4)	144 (36.5)	**< 0.001**	73 (58.4)	84 (34.6)	**< 0.001**
Allergic conjunctivitis	110 (19.9)	37 (33.0)	61 (15.5)	**< 0.001**	39 (31.2)	42 (17.3)	**0.002**
Atopic dermatitis	73 (13.2)	23 (20.5)	43 (10.9)	**0.008**	24 (19.2)	25 (10.3)	**0.017**
Food allergy	59 (10.7)	16 (14.3)	37 (9.4)	0.136	16 (12.8)	26 (10.7)	0.548
Asthma	42 (7.6)	19 (17.0)	18 (4.6)	**< 0.001**	17 (13.6)	15 (6.2)	**0.017**
Chronic urticaria	39 (7.1)	11 (9.8)	23 (5.8)	0.137	10 (8.0)	19 (7.8)	0.951
Drug allergy	29 (5.3)	11 (9.8)	14 (3.6)	**0.007**	9 (7.2)	13 (5.3)	0.478
Family history of allergy	238 (43.1)	62 (55.4)	157 (39.8)	**0.003**	65 (52.0)	87 (35.8)	**0.003**
Pet exposure place							
Home	492 (89.1)	98 (87.5)	307 (77.9)	**0.025**	73 (58.4)	93 (38.3)	**< 0.001**
Relatives' or friends' house	37 (6.7)	18 (16.1)	75 (19.0)	0.475	40 (32.0)	72 (29.6)	0.640
Workplace	17 (3.1)	11 (9.8)	42 (10.7)	0.798	6 (4.8)	26 (10.7)	0.057
Pet shop, Pet café, Pet hospital	5 (0.9)	11 (9.8)	57 (14.5)	0.203	18 (14.4)	74 (30.5)	**0.001**

Data are shown as mean ± SD or frequency (%).

∗*P*-value < 0.05 is shown as boldface in comparing variables between subjects with allergic symptoms on dog exposure and those without it or between subjects with allergic symptoms on cat exposure and those without it.

a Subjects who experienced symptoms of allergic rhinitis, allergic conjunctivitis, skin allergy, asthma and cough during exposure to dog or cat.

The study participants were aged 30.2 ± 9.2 years, and most were female (79.5%). Allergic rhinitis (44.4%) was the most common underlying allergic disease, followed by allergic conjunctivitis (19.9%), atopic dermatitis (13.2%), food allergy (10.7%), asthma (7.6%), chronic urticaria (7.1%), and drug allergy (5.3%). In total, 238 (43.1%) participants had a family history of allergic diseases. Among 506 subjects who were exposed to dogs, 112 (22.1%) experienced allergic symptoms during exposure. Among 368 individuals who were exposed to cats, 125 (33.9%) experienced allergic symptoms during exposure. There were no significant differences in sex or age between individuals with and those without allergic symptoms on dog or cat exposure. Among the study subjects who were exposed to dogs, allergic rhinitis, allergic conjunctivitis, atopic dermatitis, and asthma were more frequent among those with allergic symptoms on dog exposure than those without (71.4% vs. 36.5%, *p* < 0.001; 33.0% vs. 15.5%, *p* < 0.001; 20.5% vs. 10.9%, *p* = 0.008; 17.0% vs. 4.6%, *p* < 0.001). Similarly, among the individuals who were exposed to cats, allergic rhinitis, allergic conjunctivitis, atopic dermatitis, and asthma were more frequent among those with than those without allergic symptoms on cat exposure (58.4% vs. 34.6%, *p* < 0.001; 31.2% vs. 17.3%, *p* = 0.002; 19.2% vs. 10.3%, *p* = 0.017; 13.6% vs. 6.2%, *p* = 0.017).

Among the study participants who *were exposed to dogs,* drug allergy was more frequent among those with allergic symptoms on dog exposure than those without (9.8% vs. 3.6%, *p* = 0.007). The most common location of pet exposure was the participant's home (89.1%), followed by the home of relatives or friends (6.7%), workplaces (3.1%), and public places such as pet shops, pet cafés, and pet hospitals (0.9%). Among the subjects who were exposed to dogs, their own home was more often the place of pet exposure among those with allergic symptoms on dog exposure than those without (87.5% vs. 77.9%, *p* = 0.025). Similar findings were obtained among patients who were exposed to cats (58.4% vs. 38.3%, *p* < 0.001). Moreover, individuals with allergic symptoms on cat exposure were exposed to cats at public places less frequently compared to those without symptoms (14.4% vs. 30.5%, *p* = 0.001).

### Allergic symptoms during exposure to dogs and cats

The allergic symptoms experienced by the study participants during exposure to dogs or cats are summarized in supplemental material (Table S1). Among 112 and 125 individuals with allergic symptoms on dog or cat exposure, the most frequent allergy that occurred during exposure to the animals was allergic rhinitis (81.3% and 80.0%, respectively), followed by allergic conjunctivitis (65.2% and 73.6%), skin allergy (55.4% and 56.0%), cough (30.4% and 29.6%), and asthma (15.2% and 16.8%).

### Results of skin prick test using dog and cat dander allergen extracts

Among the 424 and 305 study participants who were exposed to dogs and cats, respectively, SPT using 3 commercially available dog and cat dander allergen extracts was conducted. As shown in Table 2, subjects with allergic symptoms on dog or cat exposure showed a significantly larger MWD and A/H ratio compared with those without symptoms, except for product B, which showed no significant difference in the MWD of dog dander allergen. The SPT positivity with 3 *reagents of dog dander allergen* was more frequently observed in individuals with allergic symptoms on dog exposure according to a criterion of any wheal, while the SPT positivity did not show consistent results by products according to other 2 criteria of MHD ≥3 mm, and A/H ratio ≥1. We found that the SPT positivity with all 3 *reagents of cat dander allergen* was more frequently observed in individuals with allergic symptoms on cat exposure according to all 3 criteria for positivity (any wheal, MHD ≥3 mm, and A/H ratio ≥1).

**Table 2:** Results of SPT using dog or cat dander allergen extracts according to allergic symptoms on dog and cat exposure.

	SPT with dog allergen (n = 424)		*P*-value∗	SPT with cat allergen (n = 305)		*P*-value∗
	Symptomatic group^a^ (n = 89)	Non-symptomatic group (n = 335)		Symptomatic group^a^ (n = 205)	Non-Symptomatic group (n = 100)	
MWD, mm						
Product (A)	1.1 ± 1.6	0.6 ± 1.3	**0.010**	2.7 ± 2.8	1.0 ± 1.9	**< 0.001**
Product (B)	0.9 ± 1.7	0.5 ± 1.3	0.061	1.4 ± 1.9	0.6 ± 1.4	**< 0.001**
Product (C)	1.7 ± 2.2	1.2 ± 1.8	**0.043**	3.1 ± 3.1	1.4 ± 2.3	**< 0.001**
A/H ratio						
Product (A)	0.3 ± 0.6	0.1 ± 0.3	**0.008**	0.6 ± 0.7	0.2 ± 0.6	**< 0.001**
Product (B)	0.2 ± 0.6	0.1 ± 0.3	**0.018**	0.2 ± 0.4	0.1 ± 0.3	**0.004**
Product (C)	0.6 ± 0.9	0.3 ± 0.5	**0.003**	0.8 ± 0.9	0.3 ± 0.6	**< 0.001**
Positivity, %						
Positive if any wheal observed						
Product (A)	29 (32.6)	60 (17.9)	**0.003**	57 (57.0)	52 (25.4)	**< 0.001**
Product (B)	24 (27.0)	54 (16.1)	**0.019**	41 (41.0)	34 (16.6)	**< 0.001**
Product (C)	41 (46.1)	123 (36.7)	**0.107**	60 (60.0)	72 (35.1)	**< 0.001**
Positive if MHD ≥3 mm						
Product (A)	23 (25.8)	42 (12.5)	**0.002**	48 (48.0)	44 (21.5)	**< 0.001**
Product (B)	15 (16.9)	39 (11.6)	0.190	30 (30.0)	26 (12.7)	**< 0.001**
Product (C)	29 (32.6)	82 (24.5)	0.122	55 (55.0)	55 (26.8)	**< 0.001**
Positive if A/H ratio ≥1						
Product (A)	8 (9.0)	13 (3.9)	0.057	26 (26.0)	16 (7.8)	**< 0.001**
Product (B)	8 (9.0)	12 (3.6)	**0.046**	10 (10.0)	6 (2.9)	**0.009**
Product (C)	25 (28.1)	43 (12.8)	**<0.001**	34 (34.0)	27 (13.2)	**< 0.001**

Data are shown as mean ± SD or frequency (%).

∗*P*-value < 0.05 is shown as boldface in comparing variables between subjects with allergic symptoms on dog exposure and those without it or between subjects with allergic symptoms on cat exposure and those without it. SPT, SPT, skin prick test; MWD, mean wheal diameter provoked by allergen in skin prick test; A/H ratio, allergen-to-histamine mean wheal diameter ratio.

a Subjects who experienced symptoms of allergic rhinitis, allergic conjunctivitis, skin allergy, asthma, and cough during exposure to dog or cat

### Serum levels of total IgE and dog/cat-dander-specific IgE

To measure serum levels of total IgE and sIgE, we collected blood samples from 454 to 331 study participants who were exposed to dogs and cats, respectively. Serum levels of total IgE were higher in individuals with allergic symptoms on dog exposure (146.60 [57.58–368.43] (median, [quartile]) vs. 71.40 [27.90–157.43] kUA/L, *p* < 0.001) and those on cat exposure (120.40 [33.40–281.70] vs. 79.85 [28.35–184.75] kUA/L, *p* = 0.043) compared with those without symptoms. Furthermore, serum levels of dog-dander-specific IgE were higher in subjects with than in those without symptoms (1.33 [0.05–7.06] vs. 0.02 [0.01–0.22] kUA/L, *p* < 0.001). Similarly, subjects with allergic symptoms on cat exposure had higher cat-dander-specific IgE levels compared with those without symptoms (1.58 [0.09–8.89] vs. 0.01 [0.00–0.42] kUA/L, *p* < 0.001; Table 3). Moreover, the number of individuals positive for dog- or cat-dander-specific IgE was higher among those with than those without allergic symptoms on dog or cat exposure; this was true for all 3 cut-off values (0.1, 0.35, or 3.5 kUA/L; *p* < 0.001 for all).

**Table 3:** Serum levels of dog- and cat-specific IgE according to allergic symptoms on dog and cat exposure.

	Dog-specific IgE (n = 454)		*P*-value∗	Cat-specific IgE (n = 331)		*P*-value∗
	Symptomatic group^a^ (n = 106)	Non-symptomatic group (n = 348)		Symptomatic group^a^ (n = 115)	Non-symptomatic group (n = 216)	
Dog or cat dander-specific IgE						
Concentration, kUA/L	1.33 [0.05–7.06]	0.02 [0.01–0.22]	**< 0.001**	1.58 [0.09–8.89]	0.01 [0.00–0.42]	**< 0.001**
Positivity, number (%)						
Positive, if sIgE ≥0.1 kUA/L	72 (67.9)	116 (33.3)	**< 0.001**	86 (74.8)	72 (33.3)	**< 0.001**
Positive, if sIgE ≥0.35 kUA/L	66 (62.3)	76 (21.8)	**< 0.001**	74 (64.3)	57 (26.4)	**< 0.001**
Positive, if sIgE ≥3.5 kUA/L	34 (32.1)	24 (6.9)	**< 0.001**	44 (38.3)	24 (11.1)	**< 0.001**

Data are shown as median [interquartile range] or frequency (%).

∗*P*-value < 0.05 is shown as boldface in comparing variables between subjects with allergic symptoms on dog exposure and those without it or between subjects with allergic symptoms on cat exposure and those without it.

a Subjects who experienced symptoms of allergic rhinitis, allergic conjunctivitis, skin allergy, asthma, and cough during exposure to dog or cat

### Diagnostic values of SPT and the sIgE level for allergic symptoms on pet exposure

The diagnostic values of SPT (dog [n = 424] and cat [n = 305]) and the sIgE level (n = 454 and n = 331) for allergic symptoms on pet exposure are shown in Table 4. The highest NPV, PPV, and SN were presented in specific IgE measurements (87.2%, 58.6%, and 67.9% for allergic symptoms on dog exposure; 83.2%, 64.7%, and 74.8% for those on cat exposure), while the highest SP were noted in SPT (96.4% for allergic symptoms on dog exposure; 97.1% for those on cat exposure). Combined sIgE and SPT results showed increased NPV and SN when at least 1 of the following 2 conditions were fulfilled: (1) a wheal of any size induced by dog and cat allergen in SPT, (2) dog and cat-dander-specific IgE level ≥ 0.01 kUA/L. They also showed increased PPV and SP when both of the following 2 conditions were fulfilled: (1) A/H ratio ≥ 1 in SPT using dog and cat allergen, (2) dog and cat-dander-specific IgE level ≥ 3.5 kUA/L.

**Table 4:** Diagnostic values of SPTs and sIgE in prediction of allergic symptoms on dog and cat exposure.

Dog allergy	PPV%	NPV%	SN%	SP%
SPT (n = 424)				
Positive if allergen provoked any wheal				
(A)	32.6	**82.1**	32.6	82.1
(B)	30.8	81.2	27.0	83.9
(C)	25.0	81.5	**46.1**	63.3
Positive if MWD ≥ 3 mm				
(A)	35.4	81.6	25.8	87.5
(B)	27.8	80.0	16.9	88.4
(C)	26.1	80.8	32.6	75.5
Positive if A/H ratio ≥ 1				
(A)	38.1	79.9	9.0	96.1
(B)	**40.0**	80.0	9.0	**96.4**
(C)	36.8	82.0	28.1	87.2
sIgE (n = 454)				
Positive if sIgE for dog dander ≥ 0.1 kUA/L	38.3	**87.2**	**67.9**	66.7
Positive if sIgE for dog dander ≥ 0.35 kUA/L	46.5	**87.2**	62.3	78.2
Positive if sIgE for dog dander ≥ 3.5 kUA/L	**58.6**	81.8	32.1	**93.1**
SPT or sIgE (n = 380)				
Positive if sIgE for dog dander ≥ 0.1 kUA/L				
or any wheal in SPT with Product A	34.7	**87.3**	68.2	63.1
or any wheal in SPT with Product B	34.5	87.0	67.1	63.4
or any wheal in SPT with Product C	29.8	87.3	**75.3**	48.8
Positive if sIgE for dog dander ≥ 3.5 kUA/L				
and A/H ratio ≥ 1 with Product A	**71.4**	78.6	5.9	**99.3**
and A/H ratio ≥ 1 with Product B	40	77.9	2.4	99.0
and A/H ratio ≥ 1 with Product C	52.9	79.1	10.6	97.3
Cat allergy	PPV%	NPV%	SN%	SP%
SPT (n = 305)				
Positive if allergen provoked any wheal				
(A)	52.3	**78.1**	**57.0**	74.6
(B)	54.7	74.3	41.0	83.4
(C)	45.5	76.9	60.0	64.9
Positive if MWD ≥ 3 mm				
(A)	52.2	75.6	48.0	78.5
(B)	53.6	71.9	30.0	87.3
(C)	50.0	76.9	55.0	73.2
Positive if A/H ratio ≥ 1				
(A)	61.9	71.9	26.0	92.2
(B)	**62.5**	68.9	10.0	**97.1**
(C)	55.7	73.0	34.0	86.8
sIgE (n = 331)				
Positive if sIgE for cat dander ≥0.1 kUA/L	54.4	**83.2**	**74.8**	66.7
Positive if sIgE for cat dander ≥0.35 kUA/L	56.5	79.5	64.3	73.6
Positive if sIgE for cat dander ≥3.5 kUA/L	**64.7**	73.0	38.3	**88.9**
Skin prick test and sIgE (n = 274)				
Positive if sIgE for cat dander ≥ 0.1 kUA/L				
or any wheal in SPT with Product A	50.3	**85.3**	**79.3**	60.4
or any wheal in SPT with Product B	51.5	84.1	76.1	63.7
or any wheal in SPT with Product C	45.3	83.2	**79.3**	51.6
Positive if sIgE for cat dander ≥ 3.5 kUA/L				
and A/H ratio ≥ 1 with Product A	56.3	67.8	9.8	96.2
and A/H ratio ≥ 1 with Product B	**71.4**	67.4	5.4	**98.9**
and A/H ratio ≥ 1 with Product C	63.0	69.6	18.5	94.5

The highest values of PPV, NPV, SN and SP in SPT, sIgE measurement and their combination are shown as boldfaces. SPT, skin prick test; PPV, Positive predictive values; NPV, negative predictive values; SN, sensitivity; SP, specificity; MWD, mean wheal diameter provoked by allergen

### Abilities of total IgE and sIgE levels and skin prick test to predict allergic symptoms on dog or cat exposure

**Fig. 2:**
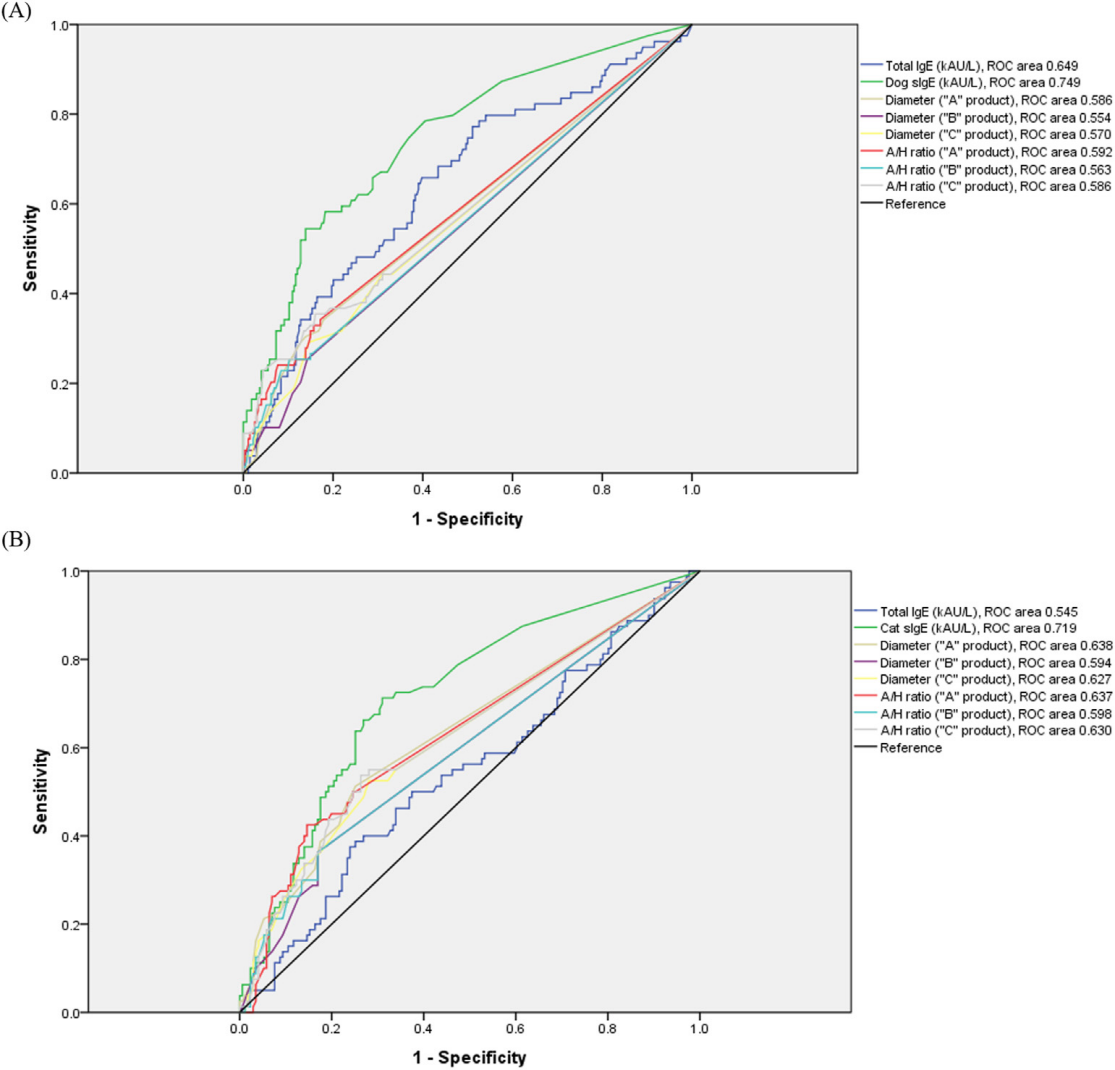
ROC curve assessments of the sensitivity and specificity of total IgE, specific IgE and SPT in predicting allergic symptoms during exposure to dog (A) and cat (B). ROC curve, receiver operating characteristic curve; SPT, skin prick test; MWD, mean wheal diameter provoked by allergen in skin prick test; A/H ratio, allergen-to-histamine mean wheal diameter ratio

The abilities of total IgE and sIgE levels and SPT to predict allergic symptoms on dog or cat exposure were assessed by ROC analysis (Fig. 2). Dog-dander-specific IgE measurements resulted in the highest AUC (0.749; 95% CI, 0.687–0.812; *p* < 0.001). When cutoff value was optimized to 0.590 kUA/L, SN and SP were 54.4% and 86.1%, respectively. Meanwhile, total IgE measurements resulted in an AUC of 0.649 (95% CI, 0.578–0.719; *p* < 0.001). Moreover, the AUC values for MWD and A/H ratio in SPT were lower than those for total IgE measurements, regardless of the allergen extract used (product A, B, or C). Similarly, cat-dander-specific IgE measurements also resulted in the highest AUC (0.719; 95% CI, 0.652–0.787; *p* < 0.001). When cutoff value was optimized to 0.085 kUA/L, SN and SP were 71.3% and 69.0%. Meanwhile, total IgE resulted in an AUC of 0.545 (95% CI, 0.467–0.622; *p* = 0.254). AUC values for MWD and A/H ratio were between those for cat-dander-specific sIgE and total IgE.

## Discussion

In this study, we performed a cross-sectional survey and evaluated the usefulness of *in vitro* assays as diagnostic tools for predicting allergic symptoms on dog and cat exposure. In Korea, pet ownership has increased drastically in recent years. In 2018, more than a quarter of all households owned pets, with dogs and cats being the most common companion animals.^27^ The increased popularity of pets could lead to an increase in allergen exposure and subsequent development of allergic diseases.

We showed that individuals with allergic symptoms during pet exposure suffered from underlying allergic conditions and frequently had a family history of allergic diseases. These results are consistent with previous studies reporting an association between pet allergies and underlying allergic diseases or atopic inheritance.^21^^,^^28^^,^^29^ We found that 22.1% and 34.0% of individuals who were exposed to dogs and cats suffered from allergic symptom on exposure, respectively; these findings are in line with previous studies reporting a 20% incidence rate of dog and cat allergies worldwide.^1^ The results of our questionnaires revealed a high frequency of self-reported symptoms, such as nasal, ocular, and skin allergic symptoms, which accounted for more than 50% of symptomatic individuals. We found that the results of SPT were variable depending on different manufacturers. Consistently, previous studies reported differences in allergenic potency and major allergen concentrations among different SPT reagents, leading to inconsistent results.^30^ Different manufacturers use different processes and raw materials to create crude extracts with uncharacterized quality and composition. Therefore, the IgE-binding capacity of these products can vary immensely.^17^^,^^31^ Importantly, a channel-activating protease inhibition study demonstrated that the total allergen potencies per protein content of dog and cat allergen extracts used in SPT differ among companies. In contrast, less variation was observed in other allergens, such as house dust mite, oak, ragweed, and Japanese hop.^17^ Furthermore, SPT reproducibility is influenced by the technique or prick test device used, as well as the criteria used to interpret skin reaction results.^12^, ^13^, ^14^ Hence, SPT suffers from low reproducibility and uncertain clinical relevance. Based on the history of exposure to pets and clinical symptoms, we found that sIgE measurements provided higher overall SN, PPV, and NPV compared with SPT. Consistently, a previous study reported that the UniCAP® (also known as ImmunoCAP®) system was more sensitive than SPT.^18^ In line with these findings, our ROC analysis suggested that dog- and cat-dander-specific sIgE levels had higher diagnostic values, with AUC values of 0.75 and 0.72, respectively. Our analyses demonstrated that dog and cat-dander-specific IgE/total IgE ratio are similar to sIgE levels in predicting self-reported allergic symptoms on dog and cat exposure (data not shown). Moreover, the AUCs for dog dander SPT were even lower than those for serum total IgE measurements, irrespective of the allergen product used in SPT. The use of ImmunoCAP® to measure circulating sIgE levels in serum or plasma can reliably identify sIgE antibody levels as low as 0.1 kUA/L. Measurement of the sIgE level using 0.1 kUA/L as the cut-off value showed high SN. Collective interpretation of SPTs (any wheal provoked by dog or cat dander allergen) or sIgE assays (dog or cat dander-specific IgE ≥0.01 kUA/L) further improved SN and NPV (75.3% and 87.3% for allergic symptoms on dog exposure**,** 85.3% and 85.3% for those on cat exposure). In contrast, another strategy using SPTs (A/H ratio ≥1 in SPT with dog or cat dander-allergen) and sIgE assays (dog or cat dander-specific IgE ≥ 3.5 kUA/L) showed very high SP (99.3% and 98.9% for allergic symptoms on dog and cat exposure, respectively) and PPV (71.4% for allergic symptoms on dog and cat exposure, respectively) but low SN (10.6% and 18.5%) and NPV (79.1% and 69.6%). Hence, with higher NPV and SN compared to SPT, serum sIgE measurements using cutoff value of 0.1 kUA/L may be more useful to exclude patients who do not warrant further investigation and who can reliably be advised that allergen avoidance is neither necessary nor helpful. When we collectively interpret results of SPT and sIgE measurement, enhanced PPV and SP may help us to identify patients with allergic symptom on pet exposure, giving a better rule-in test result.

This study has several limitations. First, our study relied on self-reported pet exposure and allergic symptoms. Thus, the possibility of reporting bias cannot be excluded, particularly for study participants whose allergies were not confirmed by doctors. Second, we could not distinguish false-positive results from asymptomatic sensitization to pet allergens. Since asymptomatic sensitization to pet allergens is a risk factor for later development of allergies, this might have introduced some bias in our results. Hence, longitudinal studies evaluating the development of pet allergies are required to confirm our findings. Third, the results of SPT and sIgE measurements reported herein might have been influenced by contamination by allergens from other sources or by cross-reactivity between dog and cat allergens.^32^, ^33^, ^34^, ^35^ Additionally, allergic symptoms can be influenced by multiple environmental factors other than exposure to allergens. Therefore, positive results in SPT or sIgE measurements do not necessarily indicate the presence of pet allergies. Provocation tests are required to confirm the presence of pet allergies. However, *provocation tests* are neither well-validated nor standardized. Additionally, allergen extract heterogeneity exists due to differences in collection site and allergen composition, compromising assay accuracy and *reproducibility, even among products from the same company.*^36^^,^^37^ Finally, study participants whose allergies were not confirmed by doctors were more likely to overestimate the actual prevalence of pet allergies.

Despite these limitations, in this study, we evaluated the presence of allergic symptoms on dog and cat exposure by comprehensively evaluating various allergic symptoms during exposure to pets, measuring serum total IgE and sIgE levels and performing SPT using 3 different commercially available allergen products. Moreover, to ensure a more accurate assessment of the diagnostic significance of these tests, we used various criteria to determine positivity in SPT and sIgE measurements and performed a collective interpretation of the two tests.

In conclusion, the measurement of dog and cat dander sIgE is more useful than SPT for the exclusion of allergic symptoms related to pet exposure. Careful interpretation of both sIgE measurements and SPT results can provide a more accurate prediction of allergic symptoms on dog or cat exposure. Considering that pet ownership and animal allergen exposure are continuously on the rise, the establishment of accurate diagnostic tests and interpretation methods can improve the diagnosis and management of dog and cat allergies.

## Ethics approval

The protocol of this study was reviewed and approved by the institutional review board (IRB approval number: GAIRB2018-194).

## Funding

This research was supported by a grant from the Korean Academy of Asthma, Allergy, and Clinical Immunology (2018), and from Basic Science Research Program through the 10.13039/501100003725National Research Foundation of Korea (NRF) funded by the 10.13039/501100002701Ministry of Education (NRF-2015R1D1A1A02061943).

## Author details

SYK, MSY, SHK and SML designed the study, collected and analyzed the data, and wrote the manuscript. SYP, JHK, HKW, OYK, JHL, YWK, JWJ, WJS and SPL contributed to the design of the study, collection and interpretation of data and revision of the draft critically for important intellectual content. All authors read and approved the final manuscript.

## Consent for publication

We hereby declare that we all participated in the study and in the development of the manuscript titled “The role of allergen-specific IgE in predicting self-reported dog and cat allergies among Korean pet exhibition participants”. We have read the final version and give our consent for the article to be published in *the World Allergy Organization Journal.*

## Availability of data and materials

The authors confirm that the data supporting the findings of this study are available within the article and its supplementary materials.

## Declaration of competing interest

The authors have no conflict of interest to declare.
