# Supplementary material for: The role of allergen-specific IgE in predicting allergic symptoms on dog and cat exposure among Korean pet exhibition participants
Source: World Allergy Organ J. 2020 Nov 27;13(12):100488. doi: 10.1016/j.waojou.2020.100488 (PMC7702179; doi:10.1016/j.waojou.2020.100488)
Supplement: Multimedia component 1 [file mmc1.doc]

**Table S1.** Allergic symptoms during exposure to dogs and cats

|  | Subjects with  allergic symptoms  on dog exposure  (n = 112) | Subjects with  allergic symptoms  on cat exposure  (n = 125) |
| --- | --- | --- |
| Allergic rhinitis | 91 (81.3) | 100 (80.0) |
| Allergic conjunctivitis | 73 (65.2) | 92 (73.6) |
| Skin allergy | 62 (55.4) | 70 (56.0) |
| Cough | 34 (30.4) | 37 (29.6) |
| Asthma | 17 (15.2) | 21 (16.8) |

Data are shown as frequency (%).
